# Static and dynamic high‐resolution ultrasound analysis of tissue distribution of poly‐L‐lactic acid particles during subdermal application in two different presentations

**DOI:** 10.1002/ski2.155

**Published:** 2022-08-19

**Authors:** Marisa Gonzaga da Cunha, Rosa Sigrist

**Affiliations:** ^1^ Department of Dermatology Faculdade de Medicina do ABC Santo André São Paulo Brazil; ^2^ Institute of Radiology Hospital das Clínicas da Faculdade de Medicina da USP São Paulo São Paulo Brazil

## Abstract

**Background:**

All the changes the skin goes through, peaking at flaccidity, occur in the dermis and hypodermis, leading to loss of support and a lower capacity to totally accommodate displacements or any loss of subjacent volume, bringing about the onset of furrows and sagging. Improvements in facial sagging may be obtained with the administrations of substances like poly‐L‐lactic acid (PLLA), which triggers a tissue response through a controlled inflammatory reaction.

**Objectives:**

Compare the tissue distribution of the particles of PLLA of both products available in Brazil (Sculptra® and Rennova Elleva®) during and immediately after their subdermal administrations, with 22G cannulas, through high‐resolution ultrasound imaging.

**Methods:**

A total of four patients aged between 18 and 64 years had the inner part of the upper arms divided into eight quadrants and treated with 16 ml of each product, reconstituted to correspond to 9.5 mg/ml. The sites where PLLA was injected were analyzed through the high‐resolution ultrasound during (dynamic imaging) and immediately after the procedures (static imaging).

**Results:**

During Sculptra® injection, high‐resolution ultrasound revealed that its distribution did not follow the trajectory of the cannula homogeneously. It was characterized by a more hyperechogenic central portion and an anechogenic peripheral portion, forming discrete posterior acoustic shadowing at times. Regarding Rennova ELLEVA® injection, the high‐resolution ultrasound analysis showed a homogeneous distribution of the product across the subcutaneous tissue following the trajectory of the cannula without formation of significant interface with the surrounding tissue, maintaining the sonographic aspect of thinly granulated hyperechogenic deposits, with strong posterior acoustic shadowing during and immediately after its administration.

**Conclusion:**

Static and dynamic high‐resolution ultrasound imaging show a more homogenous distribution of PLLA particles with the use of Rennova ELLEVA® when compared with Sculptra®, which may induce the formation of capsules and a subsequent more dispersed fibroplasia, with larger area of action and a possible better therapeutic result. The interest of this article lies in its originality, highlighting the differences in the tissue distribution of two different brands of PLLA particles, which can impact the clinical response to the two products ‐ which we are researching and seems to interfere with the increase in dermal thickness.

## Graphical abstract

Compare the tissue distribution of the particles of PLLA of both products available in Brazil (Sculptra® and Rennova Elleva®) during and immediately after their subdermal administrations, with 22G cannulas, through high‒resolution ultrasound imaging. Static and dynamic high‒resolution ultrasound imaging show a more homogenous distribution of PLLA particles with the use of Rennova ELLEVA® when compared with Sculptra®, which may induce the formation of capsules and a subsequent more dispersed fibroplasia, with larger area of action and a possible better therapeutic result.

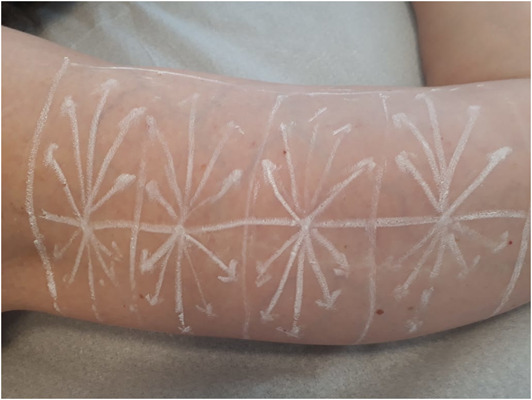

1

**What's already known about this?**

- The tissue distribution of PLLA and their differences in the two available presentations, during and immediately after application, have not yet been described or compared.

**What does this study add?**

- This study demonstrates that in the new presentation of PLLA there is a more homogeneous distribution of particles, which may lead to a better clinical response to this biostimulator for the treatment of sagging skin.

## 2 INTRODUCTION

All the changes the skin goes through, peaking at flaccidity, occur in the dermis and hypodermis. In the dermis, type I collagen fibres undergo structural changes that reduce their tension force, with higher levels of degradation and fragmentation and lower rates of fibroblast replacement, thus leading to a proportional increase in the amount of type III collagen over time. The elastic fibres gradually become frayed, without the terminal fibres that extend to the epidermis.^1^ Glycosaminoglycans and hyaluronic acid are reduced in quantity whereas the proteoglycans decorin and versican show a reduction in the molecular size of their polysaccharide chains.^2^ The enzymes responsible for the degradation of collagen and other fibres gradually increase in the skin over time, and overall, the amount of collagen per unit area in the skin decreases by approximately 1% every year.^3^ With ageing and weight loss, not only there is a reduction of fibrous septa in the hypodermis but also there are alterations in the mechanical properties of the skin, which include a progressive loss of elasticity as a longer time for the return of the skin to its original state is needed when the skin pinch test is applied.^4^ Clinically speaking, the skin becomes thinner, less tense and less elastic. As a result, there is loss of support and a lower capacity to totally accommodate displacements or any loss of subjacent volume, bringing about the onset of furrows and sagging.^2^

Improvements in facial sagging may be obtained with the administrations of substances like poly‐L‐lactic acid (PLLA), which triggers a tissue response through a controlled inflammatory reaction. The slow degradation of the material peaks with the deposition of collagen around it. Such response is subject to the characteristics of the injected product, the technique used regarding the administration of the injections in the tissue and the characteristics of each patient.^5^

Biocompatible and biodegradable, the PLLA is an injectable synthetic polymer of the alpha‐hydroxy acids family of amphiphilic nature, which forms colloidal micelles in water.^5^ The product is available in vials in the form of lyophilized powder with particles whose diameter range from 40 to 63 μm.^5^, ^6^, ^7^ The use of two brands will be compared here: Sculptra®, containing 150 mg of PLLA and 90 mg of croscarmellose sodium, a polymer with cross‐linked carboxymethyl cellulose^8^ (0.6 ratio),^6^ and Rennova ELLEVA®, containing 210 mg of PLLA with a more uniform granulometric distribution of the microparticles and 132 mg of sodium carboxymethyl cellulose (0.63 ratio).^7^ They both are emulsifying agents that keep the distribution of the particles, after the reconstitution, and the apryrogenic mannitol, which improves lyophilization.

The difference in terms of visual presentation of both products is in the lyophilization process: when it comes to Sculptra®, it happens in the vial, which makes PLLA be compressed; regarding Rennova ELLEVA®, it happens out of the vial during the preparation of the product, resulting in its presentation in powder form and making its reconstitution easier.

The aim of this study was to compare the tissue distribution of the particles of PLLA of both products during their subdermal administrations, with 22G cannulas, through a high‐resolution ultrasound imaging exam performed during the procedures.

High resolution ultrasound is a dynamic and non‐invasive imaging method that allows for the evaluation of fillers and biostimulators since such substances have their own characteristics and densities.^8^, ^9^

## 3 MATERIALS AND METHODS

### 3.1 Preparation of the products

Both vials were prepared 24 h prior to the procedures. Distilled water was used for the reconstitution, in the amount of 10 ml for Sculptra® and 14 ml for Rennova ELLEVA®, and stored under refrigeration. At administration, the products were diluted, totalling 16 ml for Sculptra® and 22.4 ml for Rennova ELLEVA®, which corresponds to a concentration of 9.37 mg of PLLA in both vials.

### 3.2 Preparation of the patients

**FIGURE 1:**
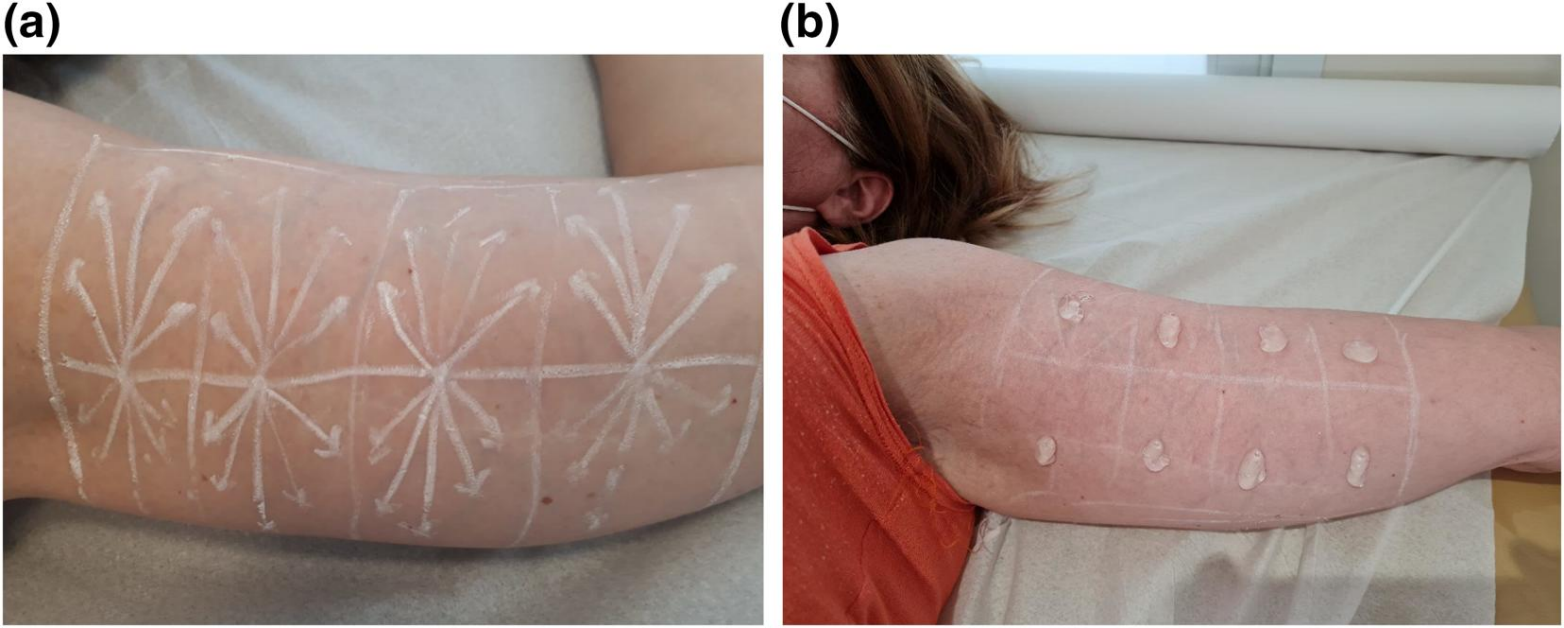
(a) Injection markings; (b) sites analyzed by high resolution ultrasound

A total of four patients aged between 18 and 64 years (mean age of 45 years) signed the free and informed consent term, which detailed the procedure itself and its objectives. Photographs were taken before and immediately after the injections. The inner part of the upper arms was divided into eight quadrants as shown in Figure 1a.

### 3.3 Administration

In each quadrant, 2 ml of the product was injected using the fanning technique (0.4 ml per retroinjection), totaling 16 ml of the product in each upper arm. Sculptra® was used in the right upper arm, whereas Rennova ELLEVA® was used in the left upper arm. Administrations were performed using the same applicator and the same technique, with Rennova cannulas 22G × 7 cm for both sides. No cannula clogging could be observed throughout the procedures.

### 3.4 Evaluation

High resolution ultrasound scan with a high frequency transducer (18 MHz) was performed with a Logiq e® scanner (GE Healthcare, Pittsburgh, PA). The sites where PLLA was injected (Figure 1b) were analyzed through the ultrasound images during the procedures (dynamic imaging) and after the procedures (static imaging, right after the procedures) by the same professional aiming to determine if there were differences regarding immediate tissue distribution of the products. Before the injections, the thickness of the complex epidermis/dermis and of the hypodermis was measured at pre‐established sites. The equipment and transducer chosen were indicated by the radiologist co‐author of this study, being at his discretion and expertise the most suitable for this purpose.

## 4 RESULTS

**FIGURE 2:**
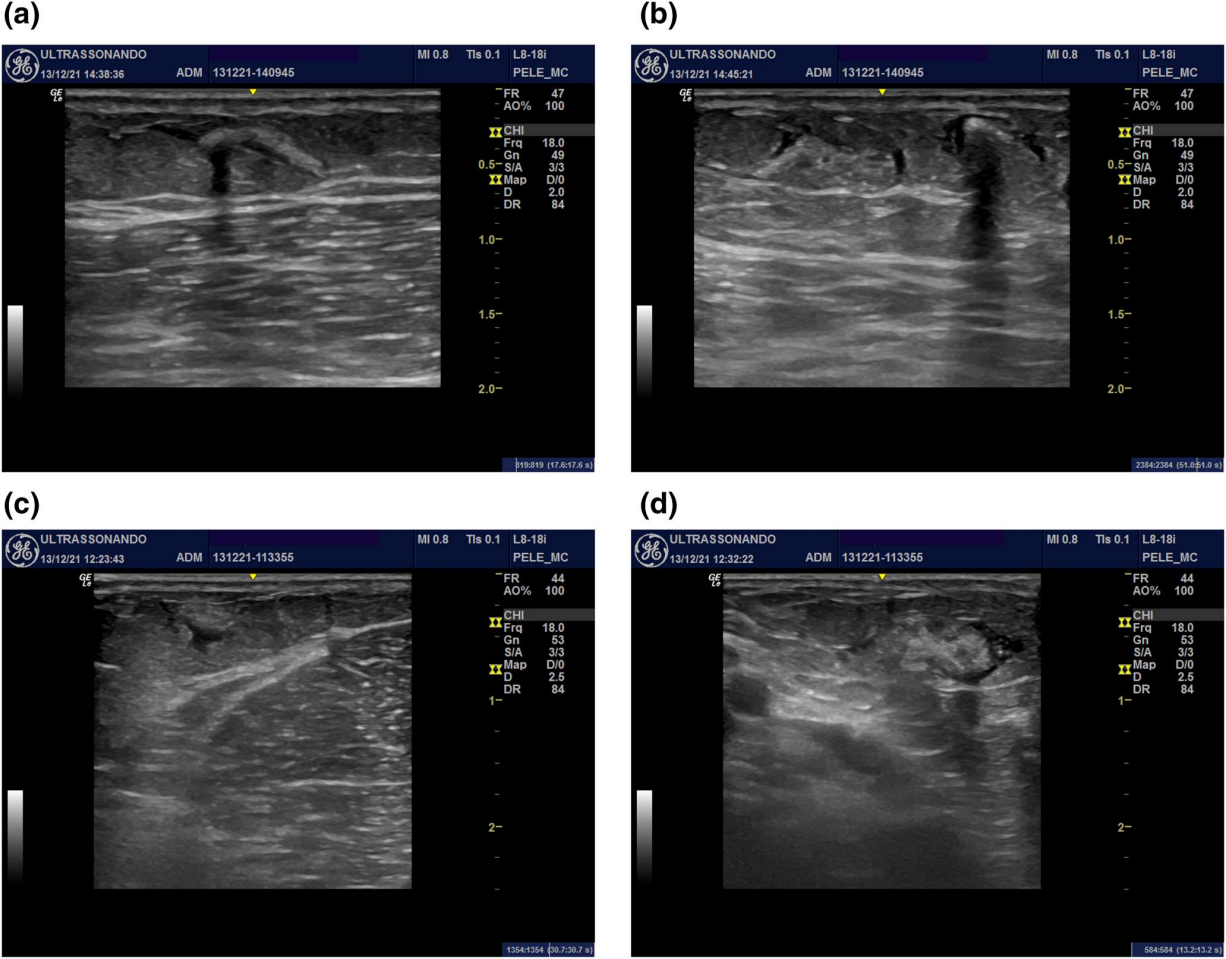
Sculptra® distribution in patient 2: (a) during; (b) immediately after the application. Sculptra® distribution in patient 3: (c) during; (d) immediately after the application

While Sculptra® was being injected, the ultrasound revealed that its distribution did not follow the trajectory of the cannula homogeneously. It was characterized by a more hyperechogenic central portion and an anechogenic peripheral portion (Figure 2a–d), forming discrete posterior acoustic shadowing at times (Figure 2a,b,2d).

**FIGURE 3:**
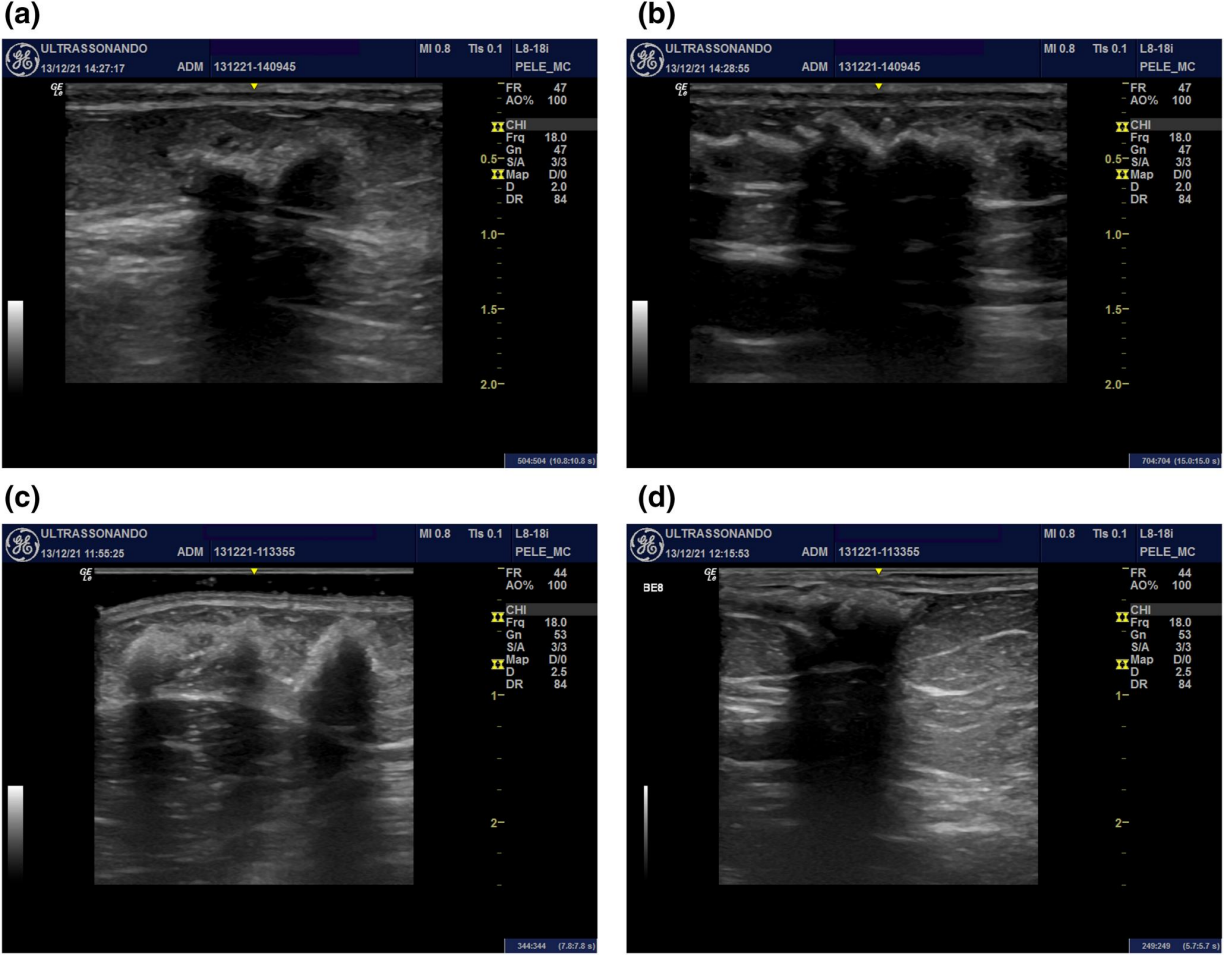
Distribution of Rennova ELLEVA® in patient 2: (a) during; (b) immediately after the application. Distribution of Rennova ELLEVA® in patient 3: (c) during; (d) immediately after the application

Figure 2a–d—Mode B ultrasound images (transverse and longitudinal views) of the inner face of the right upper arm of patients 2 (Figure 2a,b) and 3 (Figure 2c,d) showing Sculptra® distribution in the subdermal portion of the subcutaneous tissue characterized by hyperechogenic material partially wrapped in a thin anechogenic liquid layer. In Figure 2a,b, the association with discrete posterior acoustic shadowing can be observed.

Regarding the administration of Rennova ELLEVA®, the ultrasound analysis showed that during its administration there was a homogeneous distribution of the product across the subcutaneous tissue following the trajectory of the cannula without formation of significant interface with the surrounding tissue (Figure 3a–c). The sonographic aspect of the RennovaELLEVA® during and immediately after its administration is characterized by thinly granulated hyperechogenic deposits, with strong posterior acoustic shadowing (Figure 3b–d).

Figure 3a–d—Mode B ultrasound (transverse view) of the inner face of the left upper arm of patients 2 (Figures 3a,b) and 3 (Figures 3c,d) showing the Rennova ELLEVA® distribution in the superficial portion of the subcutaneous tissue. It is characterized by hyperechogenic material thinly granulated with strong posterior acoustic shadowing at the injection site.

## 5 DISCUSSION

Since 1999, injectable PLLA has been used as a cosmetic filler for the correction of skin volume loss caused by ageing. Gradually, progressively and lastingly, it promotes natural and harmonious results with low risks of adverse effects.^6^

It is a polymer with high molecular weight (140 kD) of the alpha‐hydroxy acids family, derived from the lactic acid, with properties of self‐organization and the formation of colloidal micelles in aqueous solution. These micelles act as substrate, which will promote cell activity that leads to or facilitates molecular and/or mechanical signalling so that tissue regeneration can be optimized without any local or systemic harmful response to the host.^5^

It consists of smooth‐surfaced spherical particles, with a diameter that ranges from 40 to 63 μm. The particles are *dispersed as lyophilized powder* in a sterile vial, added to carmellose sodium or to carboxymethylcellulose sodium and non‐pyrogenic mannitol. Before administration, it must be diluted in distilled water for injection, and it will be absorbed in 24–48 h.^5^, ^6^, ^10^

Two hours after the implantation of PLLA in the deep reticular dermis or the superficial hypodermis, a mild inflammatory reaction occurs in response to the foreign body, during which macrophages undergo fusion to form giant cells which try to phagocytize the particles. Edema appears to facilitate cell migration. Between seven and 10 days after the implant introduction, the macrophages secrete chemotactic and growth factors to trigger the attraction of fibroblasts and the proliferative phase of reconstruction. Fibroblasts secrete components of the extracellular matrix, initially type I collagen followed by a smaller production of type III collagen.^5^, ^6^

Fibroblasts will then isolate the implant with a fibrous collagen capsule, where each particle will be encapsulated independently from the others with substantial deposition of type III collagen close to the particles and type I collagen at the periphery of the encapsulated PLLA, thus forming mature vascularized fibrous tissue. Therefore, neocollagenesis and the consequent increase of dermal tissue, which establishes the desired cosmetic result, are obtained by marked fibroblastic activity and proliferation around each particle. It will be followed by the PLLA degradation in monomers of lactic acid through hydrolysis without any indication of acute inflammatory response.^5^, ^7^, ^11^

The mechanism of action of PLLA has important practical implications, including administration procedure, optimization of results and minimization of adverse effects.^12^ The technical differences regarding the ways PLLA is presented are small; however, they are crucial for the achievement of safe successful results.^11^, ^13^, ^14^

The lyophilization process, which is performed in the vial of Sculptra® and out of the vial of Rennova ELLEVA® during the preparation of the product, establishes important differences concerning the distribution of PLLA particles in the vial and during the reconstitution. Therefore, in order to have a more trustworthy observation of the distribution of the particles in the tissue treated with the different presentations of the product, the total volume and the amount of the product per ml were exactly the same when dilutions were carried out. It is important to point out that the amount of emulsifying agents in both fillers is practically at the same proportion, so the same amount was kept in the injected volumes.

**FIGURE 4:**
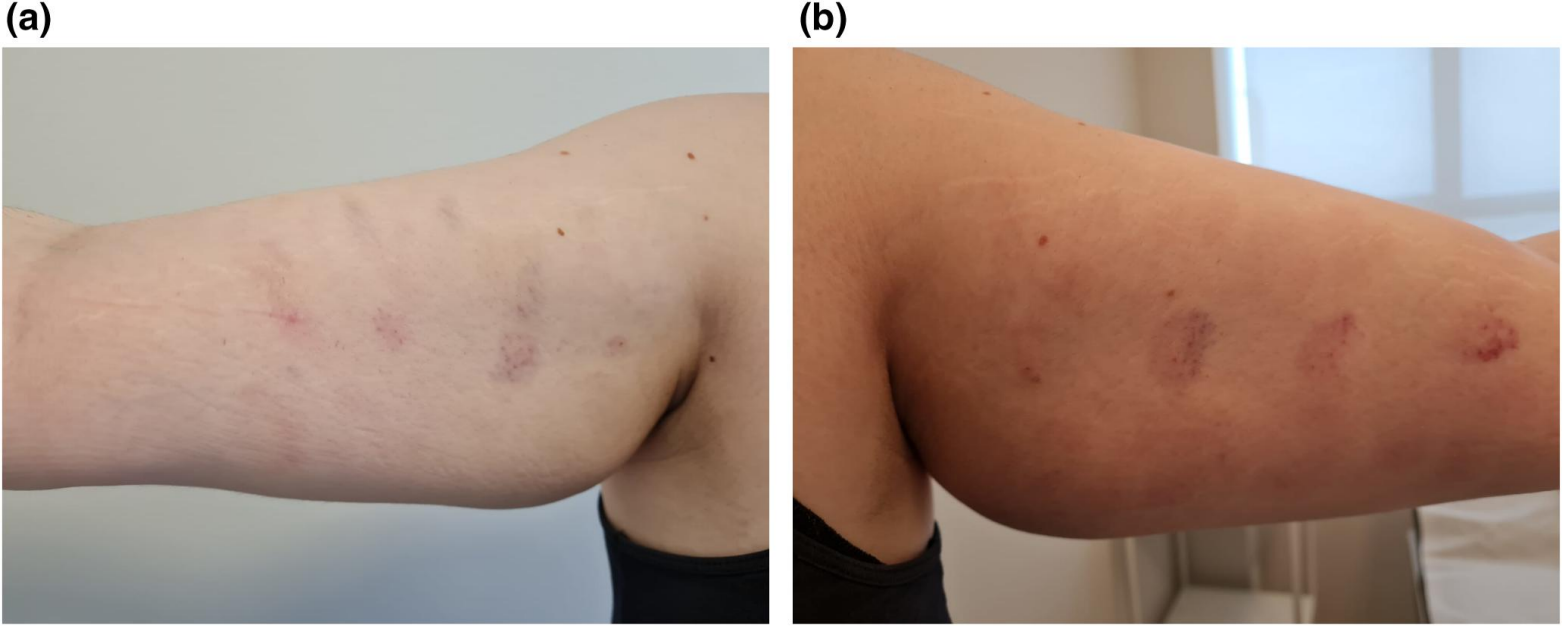
Twenty‐four hours after injection. Side 4A: Sculptra®. Side 4B: Rennova ELLEVA®

Right after the administration of PLLA, the injected volume promotes changes that can clearly be noted. Such changes may remain for 2 or 3 days until the total absorption of the diluent,^5^, ^6^ which was clinically identical between both presentations (Figure 4a,b).

There is a very small number of studies in the literature that discusses the sonographic aspect of PLLA, a solid substance to be diluted in water for injection. It is rapidly reabsorbed in up to 2 weeks after administration, leading to an increase in thickness and echogenicity of the subcutaneous tissue. Focal deposits are hardly ever observed in the application sites.^15^, ^16^ However, throughout the development of this study, which was carried out at the moment PLLA was injected, the ultrasound expression of both products here analyzed was distinct. On one hand, Sculptra® presents itself as a mildly hyperechogenic material with anechoic fluid surroundings (maybe the result of the water used for its dilution) that probably hydrodissected the fat lobules; on the other hand, Elleva® is characterized by thinly granulated hyperechogenic deposits that are well distributed by the cannula trajectory and strong posterior acoustic shadowing. Although no studies on the subject could be found in the literature, a possible explanation for the presence of posterior acoustic shadowing is the slightly higher amount of carboxymethylcellulose in Elleva®.

Despite the better distribution of Elleva® in the subcutaneous tissue through the cannula trajectory, the posterior acoustic shadow may hinder the analysis of the posterior subcutaneous tissue in response to the product. Further studies to evaluate the sonographic aspects of the different PLLA presentations throughout time should be carried out.

## 6 CONCLUSION

In the current study, high‐resolution ultrasound imaging could statically and dynamically show a more homogenous distribution of PLLA particles with the use of Rennova ELLEVA® when compared with Sculptra®. This better distribution that occurs with the former product may induce the formation of capsules and a subsequent more dispersed fibroplasia, which will promote a more effective neocollagenesis response due to the larger area of action and a possible better therapeutic result. Further comparative studies should be conducted to confirm the results found here.

## AUTHOR CONTRIBUTIONS

**Marisa Gonzaga da Cunha**: Conceptualization (equal); Data curation (equal); Formal analysis (equal); Funding acquisition (equal); Investigation (equal); Methodology (equal); Project administration (equal); Supervision (equal); Validation (equal); Visualization (equal); Writing – original draft (equal). **Rosa Sigrist**: Data curation (equal); Formal analysis (equal); Investigation (equal); Methodology (equal); Project administration (equal); Software (equal); Supervision (equal); Visualization (equal); Writing – original draft (equal).

## CONFLICTS OF INTEREST

The author declares that there is no conflict of interest that could be perceived as prejudicing the impartiality of the research reported.

## ETHICS STATEMENT

Patients signed the free and informed consent form approved by ethics committee in research, registration number 1964958.

## Data Availability

Data openly available in a public repository that issues datasets with DOIs.
